# Microdeletion and Microduplication Analysis of Chinese Conotruncal Defects Patients with Targeted Array Comparative Genomic Hybridization

**DOI:** 10.1371/journal.pone.0076314

**Published:** 2013-10-02

**Authors:** Xiaohui Gong, Xi Wu, Xiaojing Ma, Dandan Wu, Ting Zhang, Li He, Shengying Qin, Xiaotian Li

**Affiliations:** 1 Obstetrics and Gynecology Hospital of Shanghai Fudan University, the Shanghai Key Laboratory of Female Reproductive Endocrine-Related Diseases, Shanghai, China; 2 Bio-X Institutes, Key Laboratory for the Genetics of Developmental and Neuropsychiatric Disorders (Ministry of Education), Shanghai Jiao Tong University, Shanghai, China; 3 Pediatric Hospital, Shanghai Fudan University, Shanghai, China; 4 Ninth People’s Hospital, Shanghai Jiao Tong University, Shanghai, China; 5 Capital Institute of Pediatrics, Beijing, Chaoyang District, Beijing , China; Institut Jacques Monod, France

## Abstract

**Objective:**

The current study aimed to develop a reliable targeted array comparative genomic hybridization (aCGH) to detect microdeletions and microduplications in congenital conotruncal defects (CTDs), especially on 22q11.2 region, and for some other chromosomal aberrations, such as 5p15-5p, 7q11.23 and 4p16.3.

**Methods:**

Twenty-seven patients with CTDs, including 12 pulmonary atresia (PA), 10 double-outlet right ventricle (DORV), 3 transposition of great arteries (TGA), 1 tetralogy of Fallot (TOF) and one ventricular septal defect (VSD), were enrolled in this study and screened for pathogenic copy number variations (CNVs), using Agilent 8 x 15K targeted aCGH. Real-time quantitative polymerase chain reaction (qPCR) was performed to test the molecular results of targeted aCGH.

**Results:**

Four of 27 patients (14.8%) had 22q11.2 CNVs, 1 microdeletion and 3 microduplications. qPCR test confirmed the microdeletion and microduplication detected by the targeted aCGH.

**Conclusion:**

Chromosomal abnormalities were a well-known cause of multiple congenital anomalies (MCA). This aCGH using arrays with high-density coverage in the targeted regions can detect genomic imbalances including 22q11.2 and other 10 kinds CNVs effectively and quickly. This approach has the potential to be applied to detect aneuploidy and common microdeletion/microduplication syndromes on a single microarray.

## Introduction

Congenital heart diseases (CHDs) was one of the most common congenital malformation types, occurring in 5.7-7.8‰ of live births and 12.5‰ of preterm fetus [1,2]. A number of complex, multifactorial genetic and environmental influences have been cited as the causes of CHDs [3]. Copy number variations (CNVs) of chromosomal region 22q11.2 are associated with a portion of patients with CHDs. This deletion of the long arm of chromosome 22 has been found to result in DiGeorge syndrome (DGS) or Velo-cardio-facial syndrome (VCFS). There are numerous reports suggesting that 75-85% of patients suffering from the 22q11.2 deletion syndrome present CHDs; most of them are congenital conotruncal defects (CTDs) [4]. On the other hand, a substantial number of patients with CTDs have a 22q11.2 deletion [5]. Moreover, a duplication of 22q11.2 region can also lead to 22q11.2 microduplication syndrome which has features overlapping 22q11.2 deletion syndrome [6,7]. Recent evidence have suggested that infant mortality associated with CHDs has improved considerably over recent decades [8–10]. Increasing sensitivity of diagnosis means that early preparation can be made for termination, surgery therapy and psychology, with the potential to improve survival [11].

To date, various methods, including multiplex ligation-dependent probe amplification (MLPA), restriction fragment analysis on Southern blots, fluorescence in situ hybridization (FISH) and quantitative PCR (qPCR), have been used to detect disease-related genomic deletions or duplications. However, these are only capable of testing a small number of specific genes or regions [7], and occasionally may give false-positive and false-negative results.

Nowadays, comparative genomic hybridization (CGH) using oligonucleotide arrays has been implemented in cytogenetic and molecular diagnostic laboratories as a robust, rapid, sensitive, and relatively inexpensive assay for detecting various known and new gene microdeletion or microduplication [12–14]. It was first used for detecting large CNVs at the scale of multiple contiguous genes in whole genome analysis [15]. But now, more and more studies have applied targeted oligonucleotide CGH arrays because of the high-resolution and flexibility provided by these target designs [16]. We developed a targeted aCGH that permits a high-resolution analysis on Agilent platform for detecting 11 common congenital diseases, such as DiGeorge syndrome, cri du chat syndrome, Prader-Willi syndrome and so on (Table 1). Our targeted aCGH also included diseases that CNVs are rare, such as Kallma syndrome and chondrodysplasia punctata. The array was developed to detect pathogenetic microdeletions and microduplications for all the 11 congenital diseases, which are common among Chinese population, to meet our goal of offering truly comprehensive molecular testing.

**Table 1:** List of diseases diagnosed by the targeted made oligonucelotide array design.

Syndrome	Number of Probes	Chromosomal location
22q11 microdeletion/microduplication syndrome	411	22q11.2
X-Linked ichthyosis	106	Xp22.3
cri du chat syndrome	946	5p15-5p
Angelman syndrome	470	15q11-13
Miller-Dieker syndrome	302	17p13.3
Prader-Willi syndrome	470	15q11-13
Smith-Magenis syndrome	327	17p11.2
Williams syndrome	199	7q11.23
Wolf-Hirschhorn syndrome	229	4p16.3
Kallman syndrome	105	8p11.2-p12
Chondrodysplasia Punctata Type 1	200	Xp22.3

Here we described the development, validation, and implementation of a targeted, high-density oligonucleotide CGH microarray. After examining the feasibility of targeted aCGH using known cases, we tested the CNVs in postnatal patients with CTDs. In order to better understand, qPCR was used in order to confirm of CGH results.

## Materials and Methods

### Subjects

Ten cases who were already known of VCFS and 2 cases of cri du chat syndrome which deletion or duplication were confirmed by MLPA P250 kit, were tested by targeted aCGH in order to compare results of aCGH and MLPA.

Twenty-seven CTD sporadic cases (13 females and 14 males) were selected from Pediatric Hospital of Fudan University from May 2010 to June 2011. All patients had isolated CTD, the phenotypes of their parents were normal. Among the patients, there were twelve PA, ten DORV, three D-TGA, one TOF and one VSD. We chose them according to the cardiac diagnosis consistency of clinical features, echocardiography and confirmed open-heart surgery. Peripheral blood samples were obtained from these patients for analysis according to procedures approved by the Ethics Committee at Pediatric Hospital of Fudan University. In each case, the parents signed consent for our later genetic testing.

### DNA extraction

The peripheral blood samples of 27 cases and 30 healthy controls were obtained, and DNA was extracted using DNeasy Blood & Tissue Kit (Qiagen). DNAs from 30 controls were pooled and used as the control sample for aCGH and qPCR.

### aCGH protocol and data analysis

We developed a high-resolution targeted made 8x15K CGH array (Agilent customer design ID028328, Bio-X Center of Shanghai Jiao Tong University, China), which targeted known CNV regions on human chromosomes associated with 11 kinds of major genetic diseases using Agilent E-Array web tool service (https://earray.chem.agilent.com/earray/). The list of the 11 diseases was shown in Table 1. The genes that covered the 22q11 region were listed in table 2, based on the human genome release version, hg19. For oligonucleotide selection we used the probe pool provided by Agilent following recommended selection criteria for probe length, GC content and melting temperature. Sample preparation, labeling of the DNA samples, array hybridizations, scanning, image and data processing were performed according to standard protocols recommended by Agilent (Agilent, Amstelveen, Netherlands). One chip can test 8 patients one time.

**Table 2:** List of genes mapped on 22q11 region and covered by oligonucleotides designed in the targeted aCGH.

Position (HG19)	GeneSymbol	GeneName
chr22:016942359-016951255	PEX26	Homo sapiens peroxisome biogenesis factor 26
chr22:016957285-016972272	CR621131	full-length cDNA clone CS0DF030YD12 of Fetal brain of Homo sapiens
chr22:016977225-016990019	TUBA8	Homo sapiens tubulin, alpha 8
chr22:016997040-017008078	CR620426	full-length cDNA clone CS0DN004YA15 of Adult brain of Homo sapiens
chr22:017015797-017021773	USP18	Homo sapiens ubiquitin specific peptidase 18
chr22:017041724-017041773	AK129567	Homo sapiens cDNA FLJ26056 fis, clone PRS03239
chr22:017274835-017274894	DGCR6	Homo sapiens DiGeorge syndrome critical region gene 6
chr22:017280446-017304059	PRODH	Homo sapiens proline dehydrogenase (oxidase) 1
chr22:017343306-017356958	AB051434	Homo sapiens mRNA for KIAA1647 protein, partial cds
chr22:017364460-017390508	X91348	H. sapiens predicted noncoding cDNA (DGCR5)
chr22:017403824-017482073	DGCR2	Homo sapiens DiGeorge syndrome critical region gene 2
chr22:017493359-017493418	U84517	Human velo-cardio-facial syndrome 22q11 region mRNA sequence
chr22:017500055-017500143	DGCR13	Homo sapiens DiGeorge syndrome critical region gene 13
chr22:017503122-017510950	DGCR14	Homo sapiens DiGeorge syndrome critical region gene 14
chr22:017540285-017540342	CR593487	full-length cDNA clone CS0DF020YC06 of Fetal brain of Homo sapiens
chr22:017544957-017545016	SLC25A1	Homo sapiens solute carrier family 25 (mitochondrial carrier; citrate transporter), member 1
chr22:017551588-017654656	CLTCL1	Homo sapiens clathrin, heavy chain-like 1
chr22:017698213-017797435	HIRA	Homo sapiens HIR histone cell cycle regulation defective homolog A (S. cerevisiae)
chr22:017801221-017801280	MRPL40	Homo sapiens mitochondrial ribosomal protein L40
chr22:017809644-017809703	BC030758	Homo sapiens hypothetical protein LOC128977, mRNA (cDNA clone IMAGE: 4797610)
chr22:017813834-017813893	LOC128977	Homo sapiens hypothetical protein LOC128977 (LOC128977), mRNA
chr22:017818736-017839351	UFD1L	Homo sapiens ubiquitin fusion degradation 1 like (yeast) (UFD1L), transcript variant 2, mRNA

Microarray images were processed by Agilent Feature Extraction software (Agilent), and the raw data were analyzed by Agilent Genomic Workbench Lite Edition 6 5 software (Agilent). Copy number variants were identified using the aberration detection method 2 (ADM-2) statistical algorithms with a threshold of 6.0 [17,18]. ADM-2 used an iterative procedure to identify all genomic intervals with a score above a user-specified statistical threshold value (e.g., a minimum of 6 with the minimum number of probes required in a region of 3 and the minimum absolute average log ratio of 0.25). The score represented the deviation of the weighted average of the normalized log ratios from its expected value of zero and incorporates quality information about each probe measurement.

### Confirmatory test

For the deletions and duplications detected with targeted aCGH, we confirmed the breakpoints by hybridizing the DNA of the affected individuals, including 10 cases of VCFS and 2 cases of cri du chat syndrome which were diagnosised by clinical features, imageological examination or surgery and confirmed by MLPA [19].

### Real-Time PCR

Our primers were designed to lie within the exonic or intervening regions from known or putative genes using the Primer Express 3.0 software. We used the ViiA 7 Real-Time PCR System (Applied Biosystems, Darmstadt, Germany) and 384-well plates (Axygen, Union City, CA, US) for real time PCR. Reactions contained 0.25 mM each primer and 5µl Fast Start Universal SYBR Master (Roche, Basel, Switzerland) in a total of 10µl. Assays included DNA standards in a final concentration of 10.0, 2.5, 0.625, 0.15625, 0.03906 ng/µl, a no-template control, and 5 ng/µl of the patient DNA in replicates (n=3). Cycling conditions were 50°C for 2 min, 95°C for 15 min, and 40 cycles of 94°C for 15 sec, 60°C for 15 sec, and 72°C for 1 min. In order to avoid the generation of unspecific products, a melting curve analysis of products was performed routinely following the amplification. A standard curve was constructed for each amplicon by plotting the cycle number (ct) and the quantitative data were further processed to calculate the ratio relatively to the average amount of reference amplicons for each amplicons in the patients as previous described [20]. In this manner, ratio-values of 1.0 indicated diploid situation, values of 0.4-0.6 or 1.4-1.6 indicated partial haploid or partial triploid, respectively.

## Results

### Comparison of MLPA and targeted aCGH

**Figure 1:**
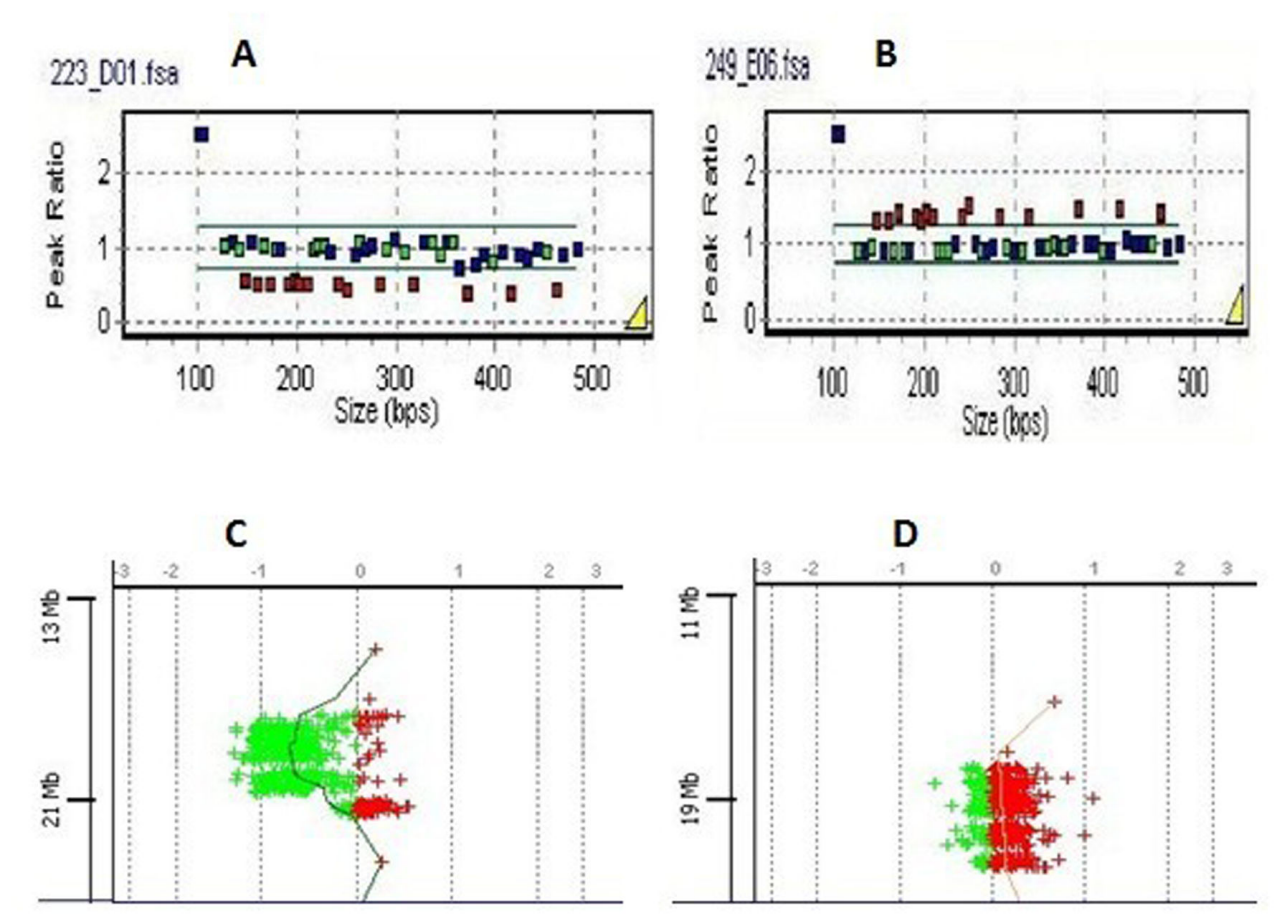
Dosage assessment of 22q11 by MLPA eletropherograms of two VCFS samples. The MLPA data was presented in a ratio analysis format where the x axes represented fragment size in bp, and the y axes represented probe-height ratios. Squares indicated either deleted probes (height ratio <0.65) or duplicated probes (height ratio>1.35). Squares located between 0.65 and 1.35 on the y axis indicated non-deleted, non-duplicated probes. Two panels showed patients’ data as follow: A represent 3 Mb 22q11.2 deletion: B represent 3 Mb 22q11.2 duplication. They were all confirmed by aCGH: gene views of 22q11 produced by the Agilent CGH Analytics software and showed the aberrant region, which was highlighted in color. The dots corresponded to the array targets, arranged on the y axes represented genomic position and on the x axes represented log_2_ intensity ratio value. C was the same case as A, D was the same case as B.

The performance of our targeted aCGH was validated by 12 known cases, including 10 cases of VCFS and 2 cases of cri du chat syndrome, which had been initially characterized by MLPA. The aCGH results were concordant with the MLPA in all 12 cases, only two cases of VCFS were shown in Figure 1.

### Comparison of targeted aCGH and qPCR

**Figure 2:**
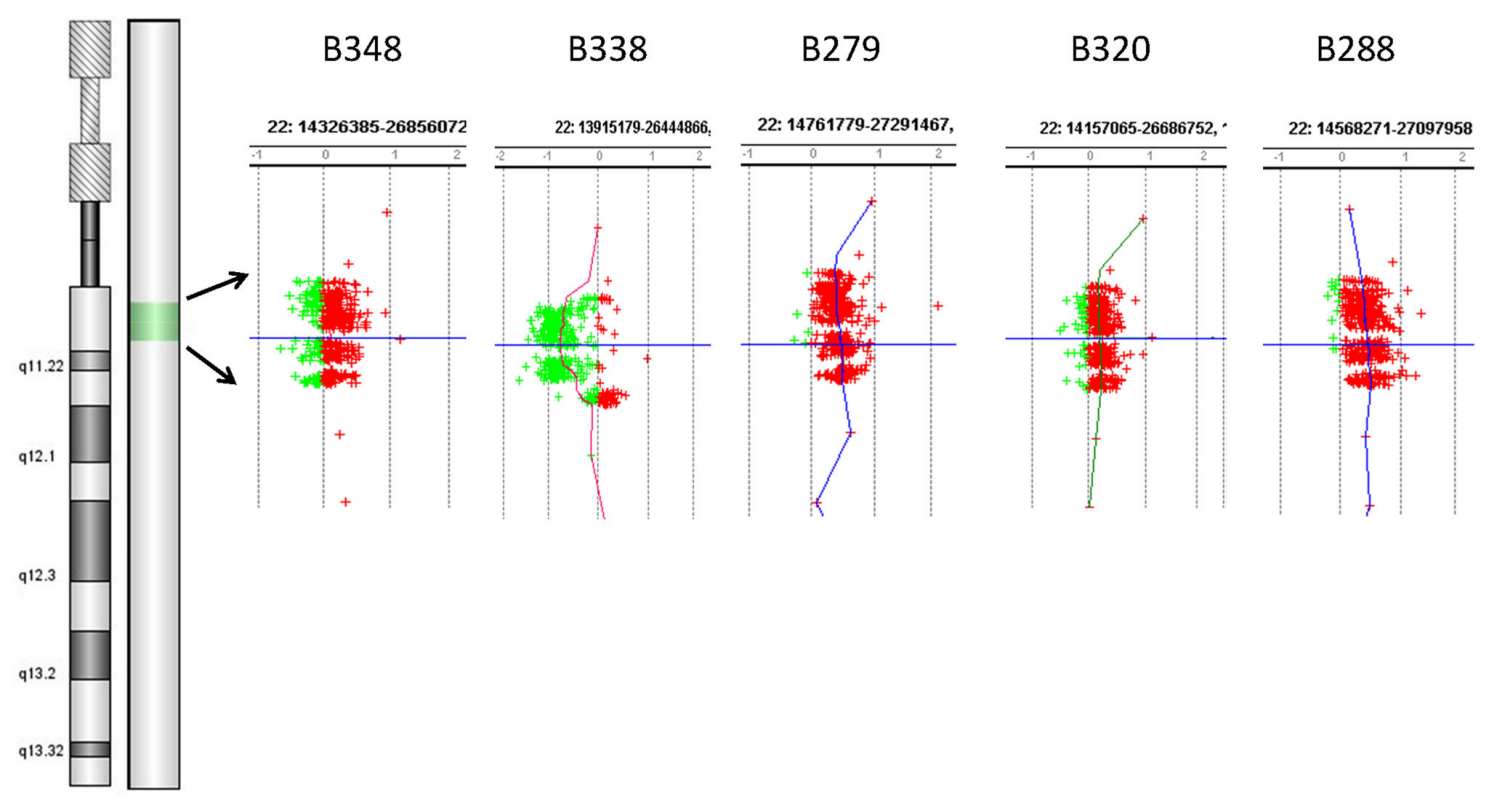
The targeted aCGH results of patient B338, B279, B320, B288. The left panel showed a whole-chromosome view of data from chromosome 22q. The Right panel showed the clustered oligonucleotide probe coverage at 22q11 genes of interest. The y axis represented genomic position of 22 chromosomes and the x axis showed the normalized log ratios. Results for oligonucleotides showing the typical copy number (log_2_ ratio=0±0.25) were shown in the middle, whereas those in green or red represent log ratios outside this range indicating copy number loss or gain, respectively.

We analyzed 27 subjects with CTDs using the Agilent 8x15K targeted CGH microarrays. It showed a heterozygous deletion of 2.6Mb on 22q11.2 region in the patient B338, and a same 3.8 Mb duplication in the patient B279, B320, B288 (Figure 2). Table 3 showed the targeted aCGH deletion and duplication results for positive samples, including size and location. aCGH results are confirmed by the qPCR (Table 4).

**Table 3:** Deletions and Duplications of 22q11.2 detected by targeted aCGH.

Patient	Gain or Loss	Location on genome (HG19)	size (bp)	
B338	Loss	18894835-21505417		2,610,582
B279	Gain	18546548-22336327		3,789,779
B320	Gain	18546548-22336327		3,789,779
B288	Gain	18546548-22336327		3,789,779

**Table 4:** Real-time PCR results.

GENE	D22S181	PRODH	TUPLE1	COMT	ZNF74	PIK4CA	LZTR1	CAT4	D22S936
START	16968759	17293217	17763150	18330640	19073892	19429805	19673422	19708684	19777314
END	16968859	17293317	17763250	18330739	19073992	19429905	19673524	19708784	19777414
B338	0.71	0.56	0.41	0.40	0.32	0.33	0.45	0.49	0.44
B279	1.41	1.53	1.50	1.54	1.42	1.58	1.49	1.33	1.58
B320	1.40	1.40	1.58	1.40	1.44	1.40	1.39	1.56	1.40
B288	1.50	1.40	1.49	1.24	1.42	1.50	1.44	1.55	1.35
C	1.03	0.99	1.05	0.95	0.97	0.97	1.01	1.03	0.99

Results of the real-time quantitative PCR applied to patient DNA samples of four cases. The normalized ratios (case amplicon/ reference amplicon) were presented. Values interpreted as haploid situation (deletion) ranged from 0.40 to 0.60, and triploid situation (duplication) ranged from 1.40-1.60, whereas a diploid situation was assumed for values from 0.90 to 1.10.

## Discussion

aCGH have been widely used to detect submicroscopic chromosomal changes, including microdeletions and microduplications across the human genome [21,22], and mitochondrial disorders [23]. Based on similar approaches, we designed, validated, and implemented a targeted aCGH analysis as an experimental molecular testing tool to simultaneously screen for deletions and duplications of eleven diseases. Targeted aCGH analysis confirmed previously characterized deletion and duplication in 12 cases with a 100% concordance. Hence, we inferred that targeted aCGH analysis can determine deletion and duplication disorders designed in the list, which some of them have CHDs.

In general, CHDs were to some extent associated with the 22q11.2 deletion and duplication [24,25]. Most kind of CHDs in patients of 22q11.2 CNVs was CTDs, about 4–6.13% of which had 22q11.2 deletion [26,27], while 0.3–0.9% of which had 22q11.2 duplication [28]. In our study the incidence of 22q11.2 CNVs was 14.8% (4/27), including 3.7% (1/27) deletion and 11.1% (3/27) duplication. We evaluated the hypothesis which suggested that 22q11.2 CNVs, may be relatively common in patients with CTDs and probably had been under-diagnosed in routine analyses, such as FISH, PCR, MLPA [29,30]. For example, FISH assay had probe for TUPLE1 or N25 on 22q11.2, but it failed to detect deletions that were either proximal or distal to the probes. These uncommon deletions were estimated to occur in 2% of 22q11.2 cases, besides, standard FISH did not provide any information about the length of the deletion. Commercially available MLPA kits was a suitable del22q11.2 screening method, but the cost and time would not meet the criteria for a population-based, primary screening tool specifically targeting patients [29]. qPCR assays depended only on several probes for each targeted region, so the efforts to examine multiple genes simultaneously have been very limited. In contrast, the availability of well-characterized targeted aCGH covering known genomic regions from established databases reduced the time to create and validate probes, and turned out to be a rapid, comprehensive, relatively inexpensive, highly sensitive, and accurate method for detecting deletions and duplications in many prenatal and postnatal malformations simultaneously on a common platform, such as 22q11 deletion and duplication syndrome [18,30], cri du chat syndrome [31] and Prader-Willi syndrome.

Because the incidences of most syndromes was low, we only found 22q11 disorder by 37 patients. Besides aneuploidy, 22q11.2 deletion is one of the most recognizable chromosomal abnormalities causing CHDs and other malformations, suggesting that prenatal genetic detection should be performed routinely for their adequate management and genetic counseling [32]. There is a similar study by Syrmou A, et al. [33] which identified submicroscopic genomic rearrangements associated with congenital heart disease (CHD) with 1x244K or 1x180K array-CGH Agilent arrays (average resolution 7-13kb). In their study, CNVs were detected in 37 of 55 CHD patients, and all the patients were reported to have at least one additional phenotypic abnormality. Moreover, unexpected genomic rearrangements in relation to CHD were identified in their study. In contrast, we performed the CGH experiments using 27 subjects with CTDs without other phenotypic features. And, we did not identify other CNVs, besides the typical 22q11.2 deletion or duplication. It may be due to that the targeted arrays are not able to identify CNVs outside the targeted regions. However, compared with the 1x244K and 4x 180K Agilent arrays, our new design can provide much higher resolution detection for these targeted regions with the average resolution of 3kb. Therefore, our study provided new evidence that targeted aCGH may appear as a promising high-resolution diagnostic tool.

In addition to its efficiency and sensitivity, this targeted CGH array is a powerful tool as it allows simultaneous analysis of several congenital disorders. Therefore, this targeted aCGH array maybe become a valuable tool for a new diagnostic approach of CNVs and could facilitate the molecular diagnosis of heterogeneous groups of diseases such as Kallman syndrome, chondrodysplasia punctata. However, we still need more clinical high index of suspected cases to test and verify other 9 kinds of diseases using our targeted aCGH.

In conclusion, the targeted aCGH is a powerful, cost-effective and fast tool for detecting pathogenetic copy number variations such as microdeletions and microduplications in known genomic region. The method can easily be used also for prenatal and postnatal diagnosis. Further more detailed study of these known regions associated with the 11 syndromes is warranted to verify our results.
